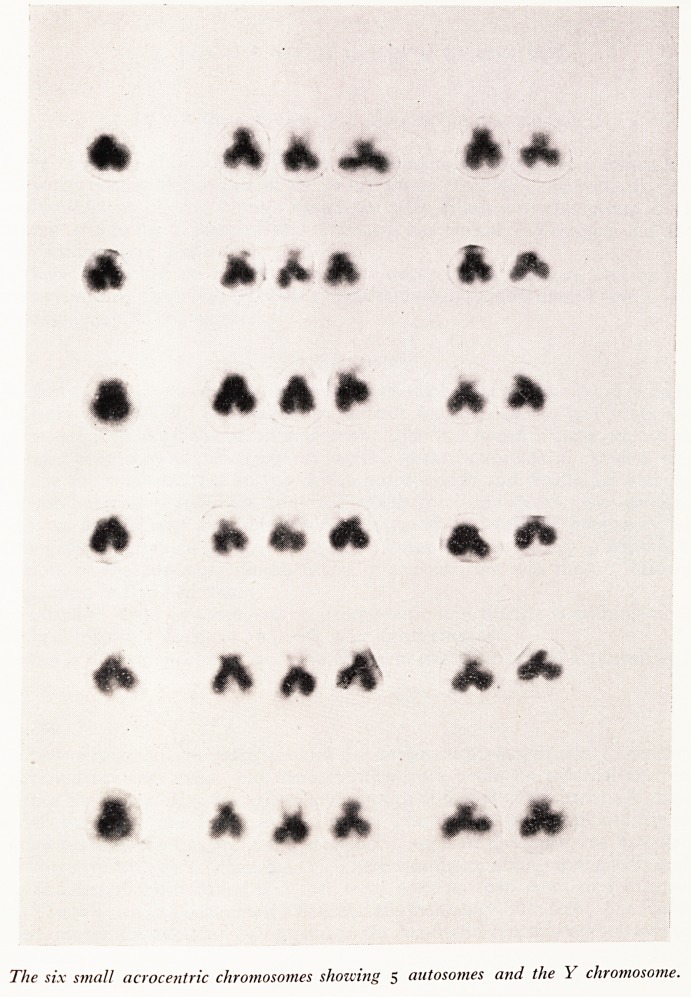# A Foetus with Down's Syndrome

**Published:** 1963-10

**Authors:** M. B. Wingate


*Ear of foetus showing flattening of upper margin.*

PLATE XXIII

*Complete karyotype*

PLATE XXIV

*The six small acrocentric chromosomes showing 5 autosomes and the Y chromosome.*

### A FOETUS WITH DOWN'S SYNDROME

BY

M. B. WINGATE, M.B., B.S., F.R.C.S. (ED.), M.R.C.O.G.

In the course of chromosome studies on spontaneous abortions, a foetus has been found with trisomy of one pair of the group 21–22 (Denver classification). To date, the only clear-cut clinical picture associated with trisomy in this group is Down's syndrome, and to try to establish this diagnosis, anatomical, radiological and dermatoglyphic studies were made.

As there have been no reports of trisomy occurring in a foetus, and only one of Down's syndrome diagnosed by clinical and dermatoglyphic studies (Thompson 1961), the findings may be of interest.

### CASE REPORT

The foetus was spontaneously aborted by a primigravida aged 30 years, who booked for delivery in hospital. She had been infertile for three and a half years and was being investigated when she became pregnant. She was found to have uterine fibroids and a mild progesterone deficiency on vaginal smear examination. At nine weeks of pregnancy she was admitted with a threatened abortion and treated by bed rest and Norethynodrel ethinyloestradiol (Envaid) tablets for three weeks. She continued to take Enavid on discharge but she was readmitted at 21 weeks of pregnancy with an inevitable abortion which became complete. There was no history of serious illness, irradiation, or the previous administration of steroids, nor was there a history of a viral infection in this pregnancy.

Her husband, aged 35 years, was in good health, and there was nothing of note in his medical history. Husband and wife were third cousins.

There was no evidence of Down's syndrome or other congenital abnormality in the family.

### *The Foetus*

The foetus weighed 431 grammes and the crown-rump length was 18 centimetres. The external sex was male. No abnormalities were noted on external examination apart from the facies. In comparison with a normal foetus of the same age there was marked flattening of the bridge of the nose. The mouth was small and the tongue protruded between the lips and the uppermost margin of the helix was flattened and turned down on both ears. (Plate XXII). There was no incurving of the fifth digit and in size and length both hands and feet were normal when compared to a normal foetus. There was a gap between the first and second toes of both feet. The foetus was dissected, particular attention being paid to the heart and central nervous system; no abnormalities were found.

Histological section of skin, testes, liver, spleen, adrenal gland, brain and lung showed no disorder of structure such as Benda (1960) described.

### *X-Ray Examination*

Radiographs were taken of the whole skeleton of the foetus and a normal foetus of the same age. There was no obvious difference in size, shape or structure of the skull, long bones or extremities, the second phalanx of the fifth digit was present in the trisomic foetus.

The iliac index of both foetuses was measured. In the trisomic it was 55°, in the normal 58.5°. (The mean index for children with Down's syndrome is 62°, and the mean normal 81°).

### *Dermatoglyphic Patterns*

The foetus had been preserved in formal saline and contracture of the skin and ligaments had taken place. It was not possible to take palm and finger prints, and various methods of photography did not give reproducible results. Direct examination under a dissecting microscope, using tangential light enabled readings to be made. Professor Penrose was kind enough to confirm and supplement the findings.

The palms both showed a single palmar crease. On the first, second, third, fourth and fifth digits of the left hand, there were ulnar loops and there was a loop pattern in the third inter-digital area. The first, second, third and fifth digits of the right hand had ulnar loops, the fourth digit had a radial loop. Again there was a loop in the third interdigital area. A hypothenar pattern with tri-radii t and t″ was present on both hands. Examination of the hallucal areas of both feet showed an open field (tibial arch) pattern. The palm, finger and foot patterns are very suggestive of Down's syndrome. (Ford Walker 1957).

### CYTOLOGICAL OBSERVATIONS

### *Nuclear Sex*

Sections of the skin and testes were fixed in Davidson's solution and stained with Thionin. No sex chromatin masses were seen in one hundred cells examined in these tissues.

### *Chromosome Studies*

Skin from the left arm and abdomen, and the right gonad were grown as explants (Harnden 1960) and good metaphase plates were obtained in all tissues from the second and third passage.

TABLE I

Summary of chromosome counts from skin of arm and abdomen and the gonad.

| | <45 | 45 | 46 | 47 | >47 |
|---|---|---|---|---|---|
| Skin of left arm .. | 12 | | 4 | 33 | |
| Skin of abdomen .. | 9 | | | 21 | |
| Gonad .. .. | 13 | 1 | 4 | 63 | 1 (49) |

The results of the chromosome counts are set out in Table I. All the cells with 45, 46 and 47 chromosomes were analysed. There was a random chromosome loss in the cells with 45 and 46 chromosomes and all had six small acrocentric chromosomes present. The cells with less than 45 chromosomes were obviously broken.

The best metaphase plates were photographed and fourteen were made into karyotypes. (Plate XXIII). Additionally a comparison partial karyotype (Plate XXIV), for the small acrocentric chromosome was made from seven cells.

In all the cells with a modal number of 46 or 47 there were six small chromosomes, one of which could be distinguished without difficulty as the Y chromosome. Examination of the five autosomes showed the presence of satellites at some time on all of them and comparison of the long arms gave the impression that the extra chromosome matched the larger of the two pairs although it was not possible to be definite about this.

*Parental Studies*

The husband was abroad and the wife was about to join him on the day following a home visit. 20 millilitres of her blood were cultured by a modification of the method of Moorhead et al. (1960), but the culture was not successful and it was only possible to count and analyse seven metaphase plates. The model number was 46 and both small and large acrocentric chromosomes were normal in number and morphology.

### DISCUSSION

The cytological findings can be interpreted as either trisomy of chromosome 21 or chromosome 22, or a fragment which, because of its satellites, can only belong to the chromosomes of group 13–15. Identification of the Y chromosome is sufficiently certain to exclude an XYY constitution. It is not possible with present techniques to distinguish between pairs 21 and 22 with certainty as both may have satellites and the long arms may appear equal.

A small number of cases with trisomy of an autosome of Group 21–22 unassociated with Down's syndrome has been described as trisomy for number 22. Hayward and Bower (1960) found trisomy 22 in a case of Sturge-Weber's syndrome, Turner and Jennings (1961) in a schizophrenic boy, Biesele et al. (1962) in two girls with a schizoid type of mental disturbance, Gustavson et al. (1962) in two severly retarded sisters with multiple malformations, and Dunn et al. (1961) in a boy with benign congential myotonia. There is therefore no clear-cut clinical syndrome associated with trisomy of Group 21–22 apart from Down's syndrome.

Trisomy of Group 13–15 is associated with such abnormalities as microcephaly, cleft palate, low set malformed ears and polydactyly (Smith et al. 1961). If the extra autosome were one of this group with deletion of part of the long arm, it is reasonable to assume that at least some of these malformations should have been present.

The disorders of growth which characterise Down's syndrome are said to be present from early intra-uterine life (Benda, 1960). Even at birth, however, all the characteristic features of Down's syndrome are not present (Carter and MacCarthy, 1951), and in the absence of any description of the findings in a foetus, a comparison cannot be made.

The facial appearance with the depressed bridge of the nose, the overlap of the ears, small oral cavity and protruding tongue, is suggestive, but the most important objective evidence is the digital, palmar and hallucal patterns. This, together with the cytological findings would strongly indicate a diagnosis of Down's syndrome.

*Acknowledgements*

The author wishes to thank Professor J. C. McClure Browne of Hammersmith Hospital, London, W.12 for providing the foetus and for permission to publish the case, Professor Penrose of University College, W.C.1. for his advice on the dermatoglyphic findings, Dr. F. Cole and Dr. R. Spector of Guy's Hospital, S.E.1 for interpreting the radiological and histological findings respectively, Mr. D. Mutton of this Unit for his work on tissue culture, Miss M. McGuire for technical assistance and Professor P. E. Polani for his advice.

This work was aided by a grant from The Spastics Society and the National Birthday Trust Fund.

### REFERENCES

Beidleman, B. (1945–6). "Mongolism in a selective review". *Amer. J. ment. Defic.* **50,** 35–53.

Benda, C. (1960). "The child with Mongolism". Grune & Stratton, New York.

Biesele, J. J., Schmid, W. and Lawlis, M. G. (1962). "Mentally retarded schizoid twin girls with 47 chromosomes". *Lancet i*, 403–405.

Carter, C. and MacCarthy, D. (1951). "Incidence of mongolism and its diagnosis in the newborn". *Brit. J. Soc. Med.* **5,** 83*90.

Dunn, H. G., Ford, D. K., Auesperg, N. and Miller, J. R. (1961). "Benign congenital hypotonia with chromosomal anomaly. "*Paediat.* **28,** 578–591.

Fraccaro, M., Kaisjer, K. and Lindsten, G. (1960). "Chromosomal abnormalities in father and mongol child". *Lancet i*, 724–7.

Gustavson, K. H., Kagberg, B., Finley, S. C. and Finley, W. H. F. (1960). "An apparently identical extra autosome in two severely retarded sisters with multiple malformations". *Cytogenetics* **1,** 32–41.

Harnden, D. G. (1960). "A human skin culture technique used for cytological examination". *Brit. J. Exp. Path.* **41,** 31–37.

Hayward, M. D. and Bower, B. D. (1960). "Chromosomal trisomy associated with the Sturge-Weber syndrome". *Lancet i*, 844–6.

Moorhead, P. S., Nowell, P. C., Mellman, W. J., Battips, D. M., and Hungerford, A. (1960). "Chromosome preparations of leucocytes cultured from human peripheral blood". *Exp. Cell Res.* **20,** 613–616.

Smith, D. W., Patau, K., Theiman, E. and Inhorn, S. L. (1960). "A new autosomal trisomy syndrome: Multiple congenital anomalies caused by an extra chromosome". *Paediat.* **57,** 338–345.

Thompson, M. W. (1961). "Reproduction in two female mongols". *Canad. J. Genet.* and *Cytol.* 3, 4, 351–354.

Turner, B. and Jenning, A. N. (1961). "Trisomy for chromosome 22". *Lancet ii*, 49–50.

Walker, N. F. (1957). "The use of dermal configurations in the diagnosis of mongolism". *J. Paediat. St. Louis.* **50,** 1, 19–20, 27–29.

## Figures and Tables

**Figure f1:**
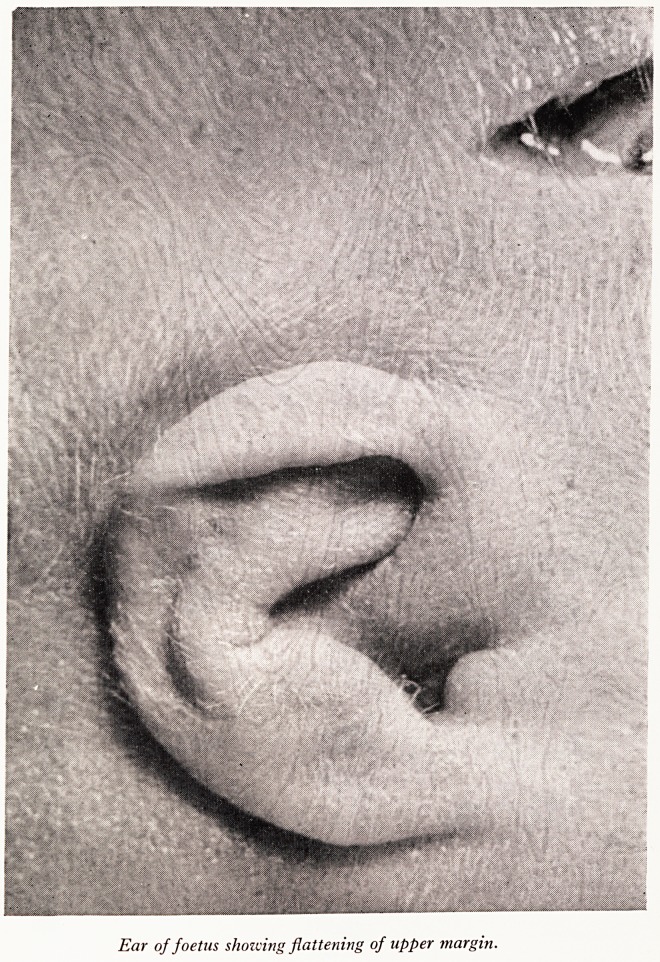


**Figure f2:**
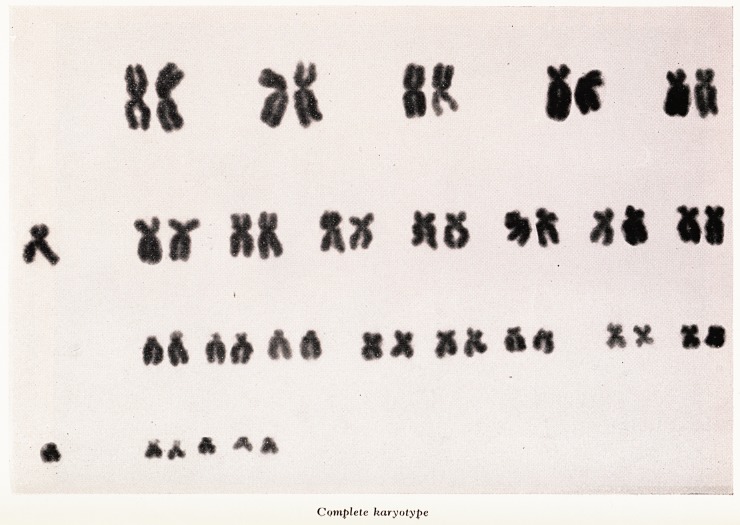


**Figure f3:**